# Autotransplanted parathyroid adenoma as a rare cause of primary hyperparathyroidism: focused literature review

**DOI:** 10.1007/s11845-026-04438-4

**Published:** 2026-05-14

**Authors:** İlgiz Tüzken, Mustafa Anıl Turhan, Ayşegül Gürsoy Çoruh, İrem Şeker, Mustafa Şahin, Volkan Genç

**Affiliations:** 1 Faculty of Medicine, Department of General Surgery, Ankara University, Ankara, 06230 Türkiye; 2 Department of General Surgery, Memorial Ankara Hospital, Ankara, 06520 Türkiye; 3 Faculty of Medicine, Department of Radiology, Ankara University, Ankara, 06230 Türkiye; 4 Faculty of Medicine, Department of Endocrinology and Metabolism, Ankara University, Ankara, 06230 Türkiye

**Keywords:** Primary hyperparathyroidism, Parathyroid neoplasm, Autologous transplantation, Muscles

## Abstract

**Background/aim:**

Primary hyperparathyroidism (PHPT) is usually caused by a parathyroid adenoma. Parathyroid autotransplantation is commonly performed during thyroidectomy when gland perfusion is impaired. However, the development of adenomas in autotransplanted tissue is extremely rare. We present a case of PHPT caused by an adenoma arising from autotransplanted parathyroid tissue, which occurred 30 years after total thyroidectomy.

**Methods:**

A 60-year-old woman with a history of total thyroidectomy and parathyroid autotransplantation to the sternocleidomastoid muscle presented with hypercalcemia and elevated parathyroid hormone levels. Ultrasonography suggested a small lesion, but sestamibi scintigraphy was negative. Definitive localization was achieved using 4D-CT and targeted ultrasonography.

**Results:**

Minimally invasive parathyroidectomy was performed, and intraoperative PTH level decreased from 318 pg/mL to 70 pg/mL. Histopathology confirmed a parathyroid adenoma originating from autotransplanted tissue. Postoperatively, calcium and PTH levels normalized.

**Conclusion:**

Adenoma formation in autotransplanted parathyroid tissue is very rare but may occur even decades after surgery. Clinicians should systematically evaluate previous autotransplantation sites when they are investigating unexplained or recurrent PHPT, even decades after thyroid or parathyroid surgery.

## Introduction

Primary hyperparathyroidism (PHPT) is most often due to a solitary parathyroid adenoma and manifests as hypercalcemia and elevated parathyroid hormone (PTH) levels [1, 2]. The parathyroid glands play a crucial role in calcium‒phosphate homeostasis. During total thyroidectomy, parathyroid glands may inadvertently be excised or devascularized, in which case parathyroid autotransplantation is a widely accepted technique to prevent permanent hypoparathyroidism, although its routine use and actual impact on long-term hypoparathyroidism remain controversial [3–5]. Although rare, hyperplasia or adenoma may develop over time in autotransplanted parathyroid tissue.

Here, we present a rare case of parathyroid adenoma arising from tissue autotransplanted into the sternocleidomastoid muscle (SCM) occurring 30 years after total thyroidectomy. This case report was prepared in accordance with the CARE guidelines. 

## Case presentation

**Fig. 1:**
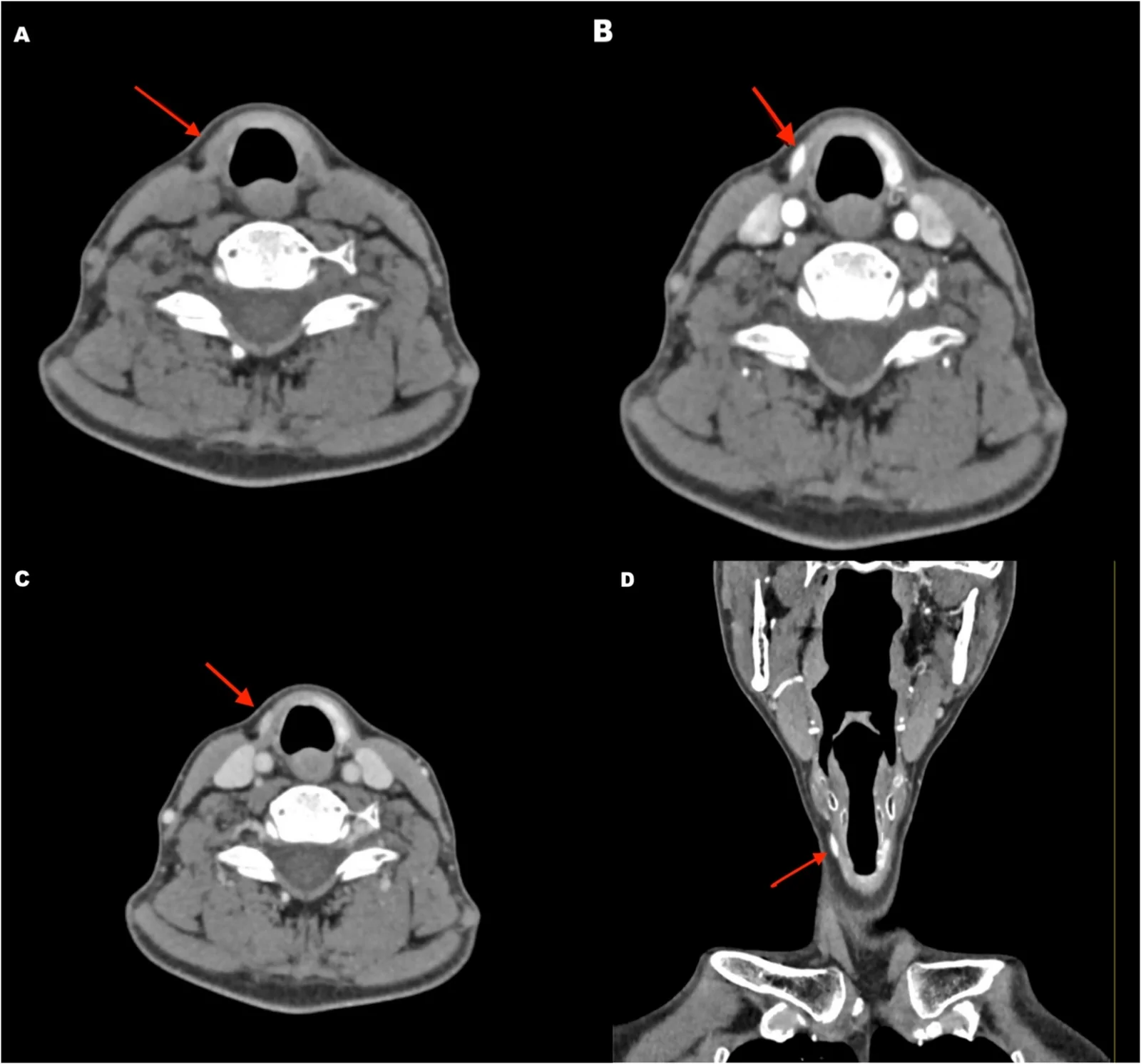
Four-dimensional contrast-enhanced CT imaging of the neck. (**A**) Non-contrast axial image shows an isodense fusiform lesion. (**B**) Axial arterial-phase images demonstrate a well-defined, avidly enhancing 7 × 5 mm lesion located anterior to the strap muscles. (**C**) The lesion exhibits a washout pattern on venous-phase images. (**D**) The arterial-phase coronal reconstructed image demonstrates an arterially hyperenhancing, tear-drop–shaped lesion located adjacent to the strap muscles, consistent with an autotransplanted parathyroid adenoma

**Fig. 2:**
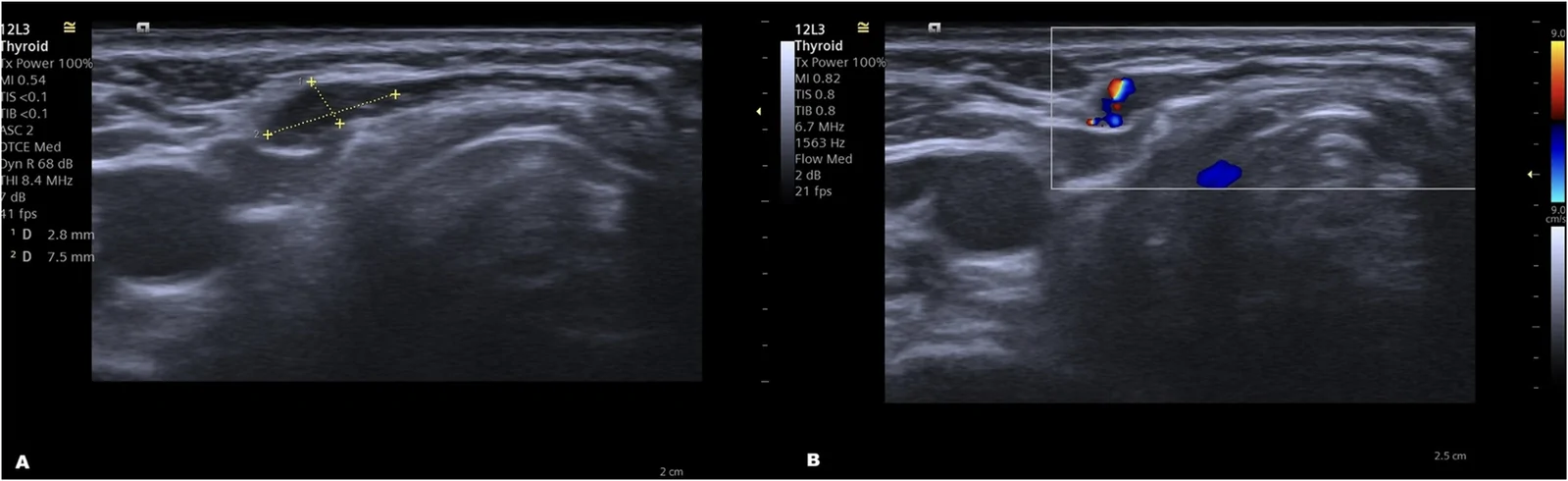
Ultrasonographic appearance of the parathyroid adenoma. (**A**) Gray-scale sonography showing a 7.5 × 2.8 mm hypoechoic nodule within the right strap muscles. (**B**) Color Doppler image revealing peripheral vascularization consistent with a functioning parathyroid lesion

A 60-year-old woman with generalized bone pain and fatigue presented at the endocrinology clinic. Her medical history included total thyroidectomy for multinodular goiter 30 years earlier and hypertension, for which she was taking levothyroxine and lercanidipine. She had no history of postoperative hypocalcemia or a need for long-term calcium/vitamin D supplementation. On physical examination, the neck was unremarkable, and her muscle strength was intact. Laboratory results were as follows: TSH 1.52 mU/L (ref. range: 0.27–4.2), T3 3.79 pmol/L (ref. range: 3.1–6.8), T4 21.7 pmol/L (ref. range: 11–22), calcium 10.9 mg/dL (ref. range: 8.6–10.2; hypercalcemia), phosphorus 2.31 mg/dL (ref. range: 2.5–4.5; hypophosphatemia), 25-hydroxyvitamin D 32.7 ng/mL (ref. range: 10–60), and PTH 318 pg/mL (ref. range: 17–74; markedly elevated). Dual-energy X-ray absorptiometry (DEXA) demonstrated advanced osteoporosis (lumbar T-score: −4.2). There was no history of nephrolithiasis or renal impairment. Initial neck ultrasonography revealed only a small 6 × 5 × 3.5 mm hypoechoic lesion in the left thyroid bed, and cytology from fine-needle aspiration was negative. Tc-99 m sestamibi scintigraphy was subsequently performed but revealed no focal tracer uptake suggestive of a parathyroid lesion. Therefore, four-dimensional contrast-enhanced computed tomography (4D-CT) of the neck was performed using a 320–detector row CT scanner (Aquilion One Genesis, Canon Medical Systems). On arterial-phase images, a hyperdense fusiform lesion measuring approximately 7 × 5 mm was identified anterior to the right strap muscles, and the lesion exhibited washout on venous-phase images (Fig. 1). Guided by these findings, a repeat targeted neck ultrasonography of this region revealed a small hypoechoic nodule in the strap muscle region with a peripheral vascularization pattern consistent with a functioning parathyroid lesion (Fig. 2).

**Fig. 3:**
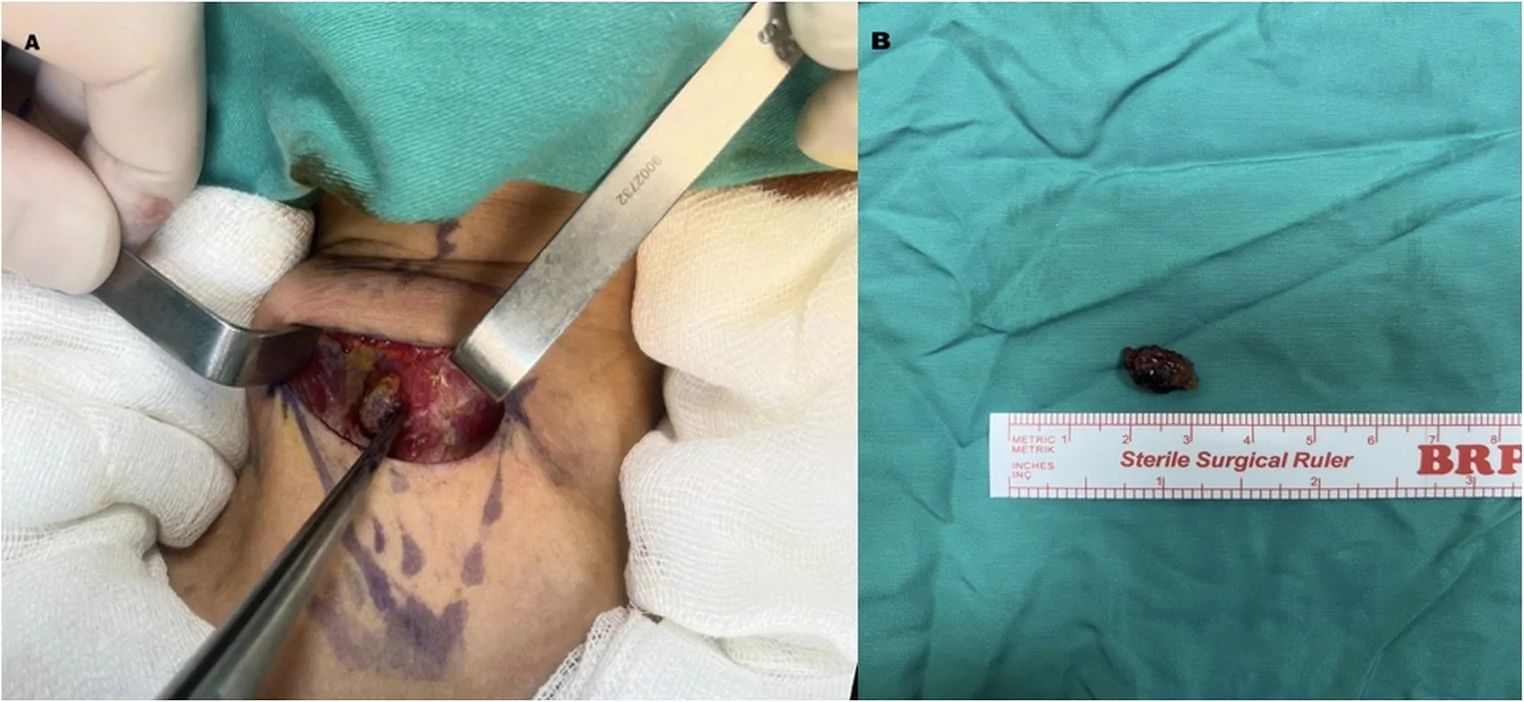
Intraoperative findings during minimally invasive parathyroidectomy. (**A**) Surgical exploration under intraoperative ultrasound guidance demonstrating the lesion within the right strap muscles. (**B**) Excised parathyroid adenoma specimen measuring approximately 1 cm, photographed next to a sterile surgical ruler

Considering these findings, a minimally invasive parathyroidectomy (MIP) was performed under intraoperative ultrasonographic guidance, and the lesion was identified adjacent to the right strap muscles, with a portion embedded within the muscle fibers (Fig. 3). Frozen sectioning confirmed that the sample was parathyroid tissue. Intraoperative PTH, measured from a peripheral venous sample 20 min after removal of the lesion, decreased from 318 pg/mL to 70 pg/mL, consistent with successful excision. Histopathologic evaluation confirmed that the sample was a parathyroid adenoma. Immunohistochemistry revealed strong nuclear positivity for parafibromin and a low Ki-67 proliferation index (≈ 1%). At 3 months of postoperative follow-up, the patient remained asymptomatic, with normalization of serum calcium and PTH levels. Further follow-up is ongoing.

## Discussion

Parathyroid autotransplantation has long been used during thyroid and parathyroid surgery with the intention of preventing permanent hypocalcemia. However, several studies have suggested that autotransplantation does not clearly reduce the risk of permanent hypoparathyroidism and may even be associated with higher rates of early or transient hypocalcemia [4–7]. Nevertheless, our case demonstrates that an autograft can remain hormonally active for decades and, in this exceptionally rare situation, give rise to a parathyroid adenoma 30 years after implantation. This illustrates that, when viable, autotransplanted tissue has the biological potential to contribute to calcium homeostasis, even if its overall impact on hypocalcemia rates remains uncertain.

Hyperfunctioning parathyroid autografts are rare and have mostly been reported in patients with secondary hyperparathyroidism (SHPT) or MEN syndromes who underwent total parathyroidectomy with autotransplantation of hyperplastic glands [8–11]. In contrast, adenomas arising from grafts that are normal or normal-appearing at the time of surgery are exceptionally uncommon. McCall and Calandra reported no recurrences among patients grafted with normal tissue [12]. To our knowledge, only a few case reports have described adenomas arising decades after autotransplantation of apparently normal parathyroid tissue [13–17], and our case represents an additional example (Table 1). It is difficult to determine whether such cases represent true neoplastic transformation of previously normal-appearing parathyroid tissue or late overgrowth of unrecognized abnormal cells.

**Table 1:** Adenoma Arising from Autotransplanted Parathyroid Tissue after Thyroidectomy

Author	Disease	Condition (at time of autotransplantation)	Site	Interval	Diagnosis	Imaging
D’Avanzo et al., [13]	PHPT (case 1)	Thyroidectomy – parathyroid autotransplantation	SCM	20 yrs	Adenoma	USG + Tc-99 m-MIBI + MRI
Harada and Otsuka, [14]	PHPT	Total thyroidectomy-parathyroid autotransplantation	Left supraclavicular region	30 yrs	No histologic confirmation	Tc-99 m-MIBI
Boggiano et al., [15]	PHPT	Near-total thyroidectomy – parathyroid autotransplantation	SCM	> 20 yrs	Adenoma	USG + Sestamibi
Thumerel et al., [16]	PHPT	Postthyroidectomy (suspected autograft) parathyroid autotransplantation	SCM	30 yrs	Adenoma	SPECT-CT + MRI + USG
MacFaul et al., [17]	PHPT	Thyroidectomy – parathyroid autotransplantation	SCM	37 yrs	Adenoma	F-18 Choline PET/CT
Current Case	PHPT	Total thyroidectomy – parathyroid autotransplantation	Right Strap Muscles	30 yrs	Adenoma	4D-CT + USG

The SCM is the most commonly used implantation site, while the brachioradialis muscle in the forearm is the second preferred option [18]. Forearm grafts are easier to assess during follow-up and, if needed, can be explored again more easily. For this reason, the brachioradialis site may be a reasonable alternative, especially in patients with a higher risk of recurrent disease, such as those with SHPT or MEN syndromes. However, as our case shows, hyperplasia or adenoma may rarely develop in long-standing autografts regardless of the implantation site. The differential diagnosis in such cases includes hyperplasia of autotransplanted tissue, adenoma arising within the graft, and residual or previously unrecognized cervical parathyroid tissue; parathyromatosis related to prior surgery may also be considered. In our patient, although the original operative note described autotransplantation into the SCM, the lesion was ultimately found adjacent to the right strap muscles and was partly embedded within the muscle fibers. Although a supernumerary ectopic parathyroid gland could theoretically be considered, the strap muscles are not an embryologically plausible site for a true ectopic gland. Therefore, in the setting of previous thyroid surgery and documented autotransplantation, the most likely explanations are either an inaccuracy in the original operative record or inadvertent implantation/seeding of parathyroid tissue during the initial surgery.

In this case, the diagnostic work-up was challenging because the lesion was located at an atypical graft site, and sestamibi scintigraphy was nondiagnostic. Ultrasonography is usually the first-line imaging modality, but its sensitivity decreases markedly in patients with ectopic or autotransplanted parathyroid tissue [19]. In contrast, owing to its high spatial resolution and characteristic enhancement pattern, 4D-CT is highly effective for localizing atypical parathyroid adenomas [20]. Functional techniques such as Tc-99 m MIBI scintigraphy and 18 F-fluorocholine PET/CT may further complement cross-sectional imaging in selected cases [17, 20]. In our patient, both ultrasonography and sestamibi scintigraphy failed to identify the lesion, whereas 4D-CT clearly demonstrated an arterially enhancing nodule embedded within the right strap muscles, allowing definitive localization and guiding repeated targeted neck ultrasonography, which confirmed the lesion. These findings support the use of 4D-CT early in the diagnostic algorithm when graft-related disease is suspected in patients with a history of thyroid or parathyroid surgery and highlight that targeted follow-up ultrasonography based on cross-sectional imaging should be considered a simple, widely available adjunct in similar cases.

Another important consideration is the need to thoroughly review the surgical history of PHPT patients in whom an adenoma cannot be localized. Careful examination of prior operative records is essential to determine whether parathyroid tissue was autotransplanted and to identify the exact implantation site, as this information is crucial for targeted imaging and surgical planning. Additionally, if autotransplantation is performed, marking the implantation site during the initial surgery may facilitate future localization and prevent unnecessary re-exploration [13].

Histopathologic findings in our case were consistent with those of previous reports of graft-derived adenomas. Adenomas arising from autotransplanted parathyroid tissue have generally been described as typical benign lesions, without any distinctive morphological features compared with conventional parathyroid adenomas [15, 17]. In accordance with these reports, the lesion in our case also showed a benign histologic appearance, with a low Ki-67 proliferation index and preserved parafibromin expression.

## Conclusion

Adenoma arising from autotransplanted parathyroid tissue is an exceedingly rare cause of primary hyperparathyroidism and may present several decades after the initial thyroid surgery. Clinicians should remain vigilant and systematically evaluate potential autograft sites in patients who present with hypercalcemia and elevated PTH levels long after thyroidectomy. A thorough review of prior operative reports, together with the early use of advanced imaging modalities, is essential for accurate localization. Rapid intraoperative PTH monitoring and meticulous surgical exploration remain critical components of definitive management.

## Data Availability

The data supporting the findings of this case report are contained within the article. Further data are available from the corresponding author upon reasonable request.
